# *Protein tyrosine phosphatase non-receptor 22* and *C-Src tyrosine kinase* genes are down-regulated in patients with rheumatoid arthritis

**DOI:** 10.1038/s41598-017-10915-9

**Published:** 2017-09-05

**Authors:** Sara Remuzgo-Martínez, Fernanda Genre, Santos Castañeda, Alfonso Corrales, Pablo Moreno-Fresneda, Begoña Ubilla, Verónica Mijares, Virginia Portilla, Jesús González-Vela, Trinitario Pina, Gonzalo Ocejo-Vinyals, Juan Irure-Ventura, Ricardo Blanco, Javier Martín, Javier Llorca, Raquel López-Mejías, Miguel A. González-Gay

**Affiliations:** 1 Epidemiology, Genetics and Atherosclerosis Research Group on Systemic Inflammatory Diseases, IDIVAL, Santander, Spain; 2 Rheumatology Division, Hospital Universitario La Princesa, IIS-IP, Madrid, Spain; 3 Immunology Division, Hospital Universitario Marqués de Valdecilla, Santander, Spain; 4 Instituto de Parasitología y Biomedicina “López-Neyra”, CSIC, PTS Granada, Granada, Spain; 5 Department of Epidemiology and Computational Biology, School of Medicine, University of Cantabria, and CIBER Epidemiología y Salud Pública (CIBERESP), IDIVAL, Santander, Spain; 6 School of Medicine, University of Cantabria, Santander, Spain

## Abstract

Several *protein tyrosine phosphatase non-receptor 22* (*PTPN22)* single-nucleotide polymorphisms (SNPs) have been significantly related with rheumatoid arthritis (RA) susceptibility. Nevertheless, its potential influence on *PTPN22* expression in RA has not been completely elucidated. Furthermore, PTPN22 binds to C-Src tyrosine kinase (CSK) forming a key complex in autoimmunity. However, the information of *CSK* gene in RA is scarce. In this study, we analyzed the relative *PTPN22* and *CSK* expression in peripheral blood from 89 RA patients and 43 controls to determine if the most relevant *PTPN22* (rs2488457, rs2476601 and rs33996649) and *CSK* (rs34933034 and rs1378942) polymorphisms may influence on *PTPN22* and *CSK* expression in RA. The association between *PTPN22* and *CSK* expression in RA patients and their clinical characteristics was also evaluated. Our study shows for the first time a marked down-regulation of *PTPN22* expression in RA patients carrying the risk alleles of *PTPN22* rs2488457 and rs2476601 compared to controls (p = 0.004 and p = 0.007, respectively). Furthermore, *CSK* expression was significantly lower in RA patients than in controls (p < 0.0001). Interestingly, a reduced *PTPN22* expression was disclosed in RA patients with ischemic heart disease (p = 0.009). The transcriptional suppression of this PTPN22/CSK complex may have a noteworthy clinical relevance in RA patients.

## Introduction

Mutations in the *protein tyrosine phosphatase non-receptor 22* (*PTPN22*) gene are associated with numerous autoimmune diseases^1, 2^. In this regard, several *PTPN22* single-nucleotide polymorphisms (SNPs) have been significantly related with susceptibility to rheumatoid arthritis (RA)^3–7^. Moreover, it has been suggested that *PTPN22* modulation, at a transcriptional level, may influence on inflammatory processes associated with RA^8, 9^. In particular, the expression of a *PTPN22* isoform was correlated with RA activity in whole peripheral blood from RA patients^10^. Accordingly, *PTPN22* is considered as the main non-HLA genetic risk factor involved in RA pathogenesis^11^, and, in fact, *PTPN22* expression profiles have been proposed as biomarkers of RA^8–10^. Despite these evidences, the influence of *PTPN22* genetic variants on *PTPN22* expression in RA has not been completely elucidated and contradictory results have been published. In this regard, whereas a study did not find any polymorphism associated with the expression of *PTPN22* splice forms in peripheral blood cells of RA patients^8^, a possible trend for association between *PTPN22* rs2488457 SNP and *PTPN22* gene expression was described in RA patients from China^12^.

Since PTPN22 is an intracellular tyrosine phosphatase that mainly inhibits T-cell receptor (TCR) signaling pathway, it is critically involved in the development of autoimmune diseases^1, 2^. This function is strengthened by the interaction with C-Src tyrosine kinase (CSK), also a negative regulator of TCR signaling^13, 14^. In this regard, two well-known *CSK* SNPs (rs34933034 and rs1378942) have been linked to systemic lupus erythematosus and systemic sclerosis^15, 16^. However, to the best of our knowledge, the information of *CSK* gene in RA is scarce.

A recent work from Walsh *et al*. reported some *PTPN22* and *CSK* polymorphisms as potential expression quantitative trait loci (eQTLs) in whole blood from RA patients^17^ (information retrieved from additional files included in that manuscript). However, this study was not specially focused on the implication of *PTPN22* and *CSK* in the pathogenesis of RA.

Taking all these considerations into account, in this study we determined if the most relevant *PTPN22* (rs2488457, rs2476601 and rs33996649) and *CSK* (rs34933034 and rs1378942) polymorphisms may influence on *PTPN22* and *CSK* expression in whole peripheral blood of RA patients when compared to healthy controls. The association between *PTPN22* and *CSK* expression in RA patients and their clinical characteristics was also studied.

## Results

### Influence of *PTPN22* genetic variants on *PTPN22* expression

**Figure 1:**
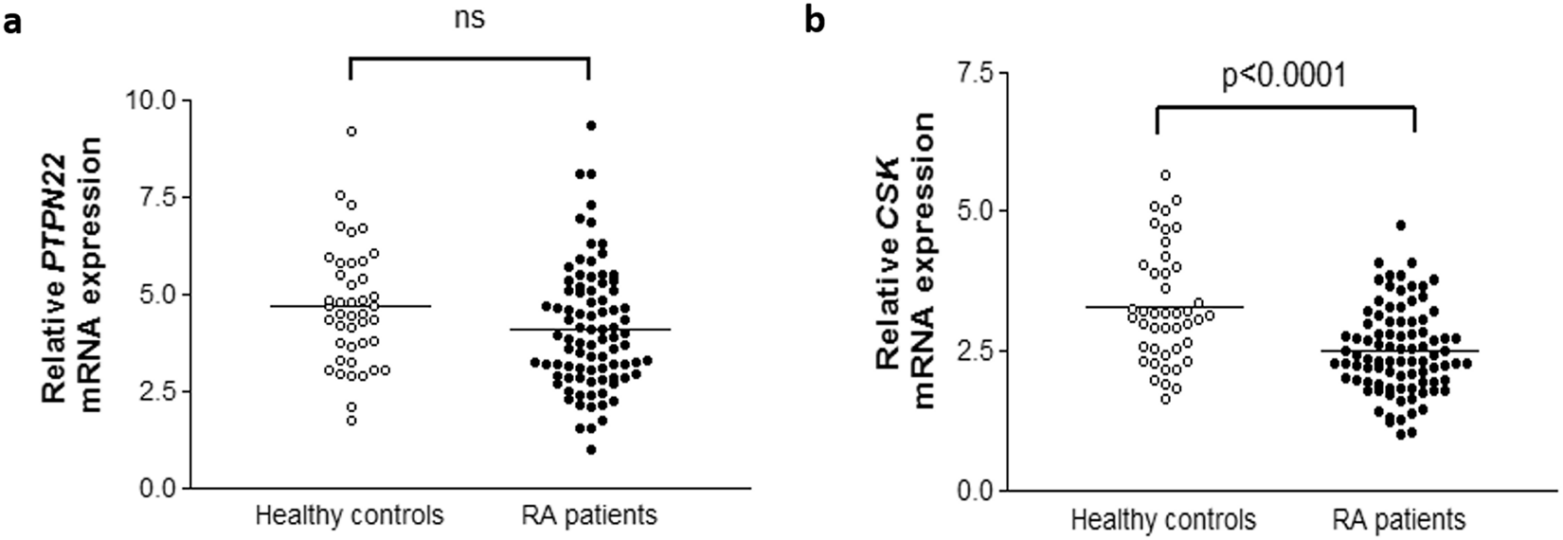
Relative *PTPN22* (**a**) and *CSK* (**b**) mRNA expression in healthy controls and patients with RA. *PTPN22* and *CSK* expression was normalized to *beta-actin* and *GAPDH* as housekeeping genes. P-values were adjusted for sex, age, and cardiovascular risk factors. Horizontal bars indicate mean value of each study group. ns indicates not significant differences.

In a first step, we compared the *PTPN22* mRNA expression between healthy controls and patients with RA (4.66 ± 1.52 vs 4.07 ± 1.57, p = 0.04). However, these differences were not statistically significant after adjustment (p = 0.22) (Fig. 1a).

**Figure 2:**
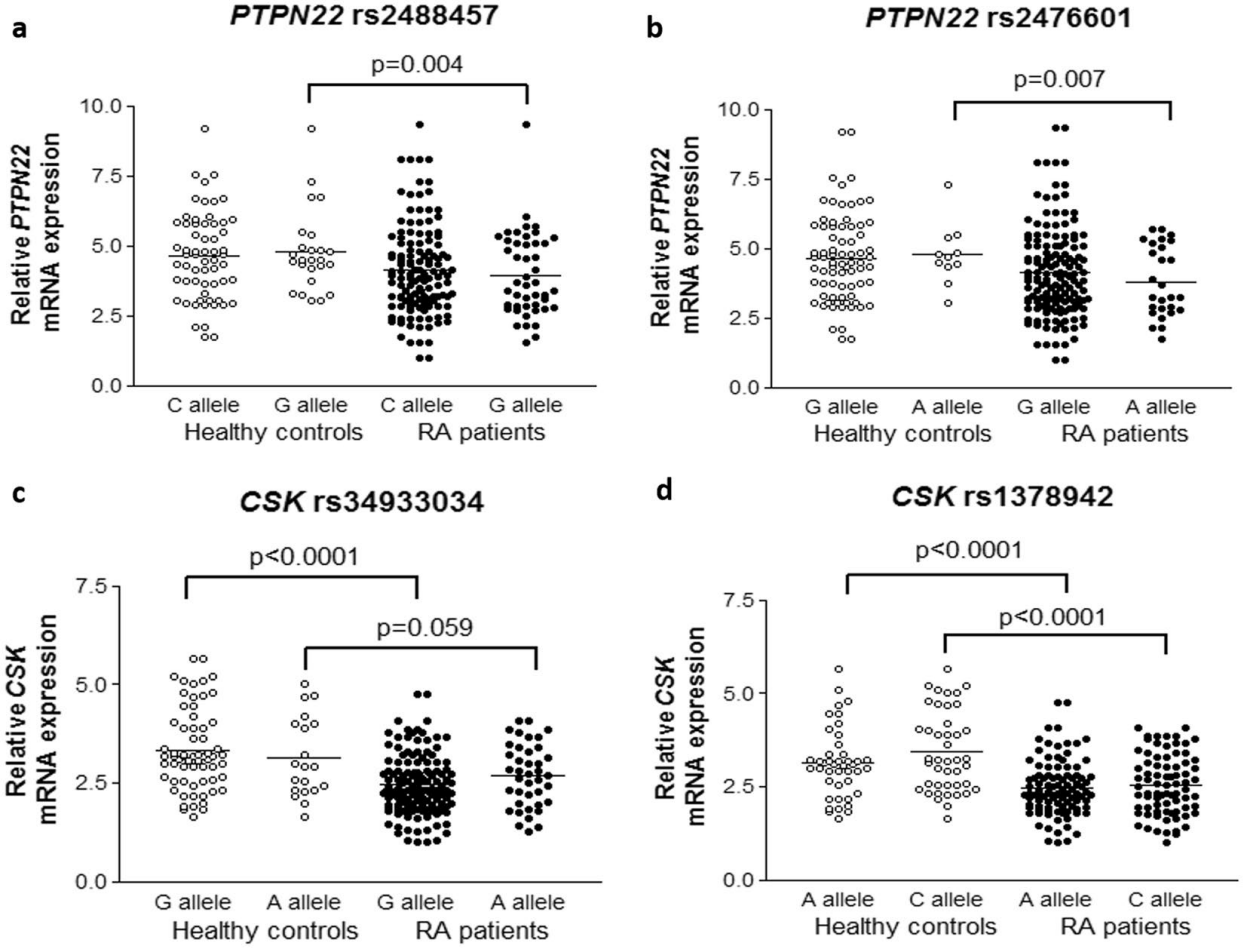
Relative *PTPN22* and *CSK* mRNA expression in healthy controls and patients with RA according to *PTPN22* and *CSK* polymorphisms. *PTPN22* and *CSK* expression was normalized to *beta-actin* and *GAPDH* as housekeeping genes. P-values were adjusted for sex, age, and cardiovascular risk factors. (**a** and **b**) Differential *PTPN22* expression between controls and patients with RA stratified according to their reference and risk allele of *PTPN22* rs2488457 (**a**) and *PTPN22* rs2476601 (**b**) polymorphisms. (**c** and **d**) Differential C*SK* expression between controls and patients with RA stratified according to their reference and risk allele of *CSK* rs34933034 (**c**) and *CSK* rs1378942 (**d**) polymorphisms. Horizontal bars indicate mean value of each study group.

To determine if *PTPN22* rs2488457, rs2476601 and rs33996649 polymorphisms may influence on *PTPN22* expression, we assessed the *PTPN22* expression in RA patients and controls carrying the reference and the risk allele for each polymorphism (Table 1). After checking that *PTPN22* rs2488457, rs2476601 and rs33996649 were in HWE, we observed a significant down-regulation of *PTPN22* in patients with RA carrying *PTPN22* rs2488457 risk allele G and rs2476601 risk allele A compared to healthy controls (p = 0.004 and 0.007, respectively) (Fig. 2a,b and Table 1 **)**. A decreased *PTPN22* mRNA expression was also disclosed between RA patients and healthy controls carrying the haplotype GA, composed of both risk alleles (Supplementary Table 1). No statistical analysis could be carried out to compare *PTPN22* expression between patients and controls carrying the *PTPN22* rs33996649 polymorphism risk allele T, since only one control subject carried this allele (Table 1). According to individuals carrying the reference allele, no significant differences in *PTPN22* mRNA expression were found between patients with RA and controls in any of the cases (Table 1).

**Table 1:** Differential *PTPN22* mRNA expression between healthy controls and RA patients according to their reference or risk allele for each *PTPN22* SNP.

SNP	Allele	Healthy controls n (%) n = 86	RA patients n (%) n = 174	*PTPN22* mRNA expression controls vs RA patients (mean ± SD)	P-value*
***PTPN22*** **rs2488457**	C	61 (70.9)	128 (73.6)	4.62 ± 1.55 vs 4.11 ± 1.60	0.89
	G	25 (29.1)	46 (26.4)	4.78 ± 1.44 vs 3.95 ± 1.48	**0.004**
***PTPN22*** **rs2476601**	G	75 (87.2)	147 (84.5)	4.65 ± 1.57 vs 4.12 ± 1.62	0.47
	A	11 (12.8)	27 (15.5)	4.76 ± 1.07 vs 3.80 ± 1.26	**0.007**
***PTPN22*** **rs33996649**	C	84 (97.7)	167 (96.0)	4.66 ± 1.53 vs 4.07 ± 1.56	0.09
	T	2 (2.3)	7 (4.0)	4.74 ± 0.07 vs 4.03 ± 1.96	—

RA: rheumatoid arthritis, SNP: single-nucleotide polymorphism, SD: standard deviation. Results in bold show statistically significant diffferences (p < 0.05). *P-values were adjusted by sex, age, and cardiovascular risk factors.

### Influence of *CSK* genetic variants on *CSK* expression

Firstly, we found a significant decrease in *CSK* mRNA expression in RA patients compared to controls (2.50 ± 0.77 vs 3.28 ± 1.02, p < 0.0001) (Fig. 1b). Then, we compared the *CSK* expression between these two study groups according to their reference and risk allele for *CSK* rs34933034 and rs1378942 (Table 2). Both polymorphisms were in HWE. We disclosed a decreased expression of *CSK* in patients with RA respect to controls, independently on whether they carried the *CSK* rs34933034 and rs1378942 reference or risk allele (Fig. 2c,d and Table 2 **)**. This decrease in *CSK* expression was statistically significant in all the cases (p < 0.05), except for individuals who carried *CSK* rs34933034 risk allele A, that showed a marginally significant decrease (p = 0.059). Similar results were obtained when haplotype analysis was performed (Supplementary Table 1).

**Table 2:** Differential *CSK* mRNA expression between healthy controls and RA patients according to their reference or risk allele for each *CSK* SNP.

SNP	Allele	Healthy controls n (%) n = 86	RA patients n (%) n = 178	*CSK* mRNA expression controls vs RA patients (mean ± SD)	P-value*
***CSK*** **rs34933034**	G	64 (76.2)	138 (77.5)	3.33 ± 1.03 vs 2.45 ± 0.75	**<0.0001**
	A	20 (23.8)	40 (22.5)	3.14 ± 1.01 vs 2.69 ± 0.79	0.059
***CSK*** **rs1378942**	A	43 (50.0)	100 (56.2)	3.13 ± 0.93 vs 2.47 ± 0.74	**<0.0001**
	C	43 (50.0)	78 (43.8)	3.44 ± 1.07 vs 2.55 ± 0.80	**<0.0001**

RA: rheumatoid arthritis, SNP: single-nucleotide polymorphism, SD: standard deviation. Results in bold show statistically significant diffferences (p < 0.05). *P-values were adjusted by sex, age, and cardiovascular risk factors.

### Association of *PTPN22* and *CSK* mRNA expression with clinical characteristics in RA patients

**Figure 3:**
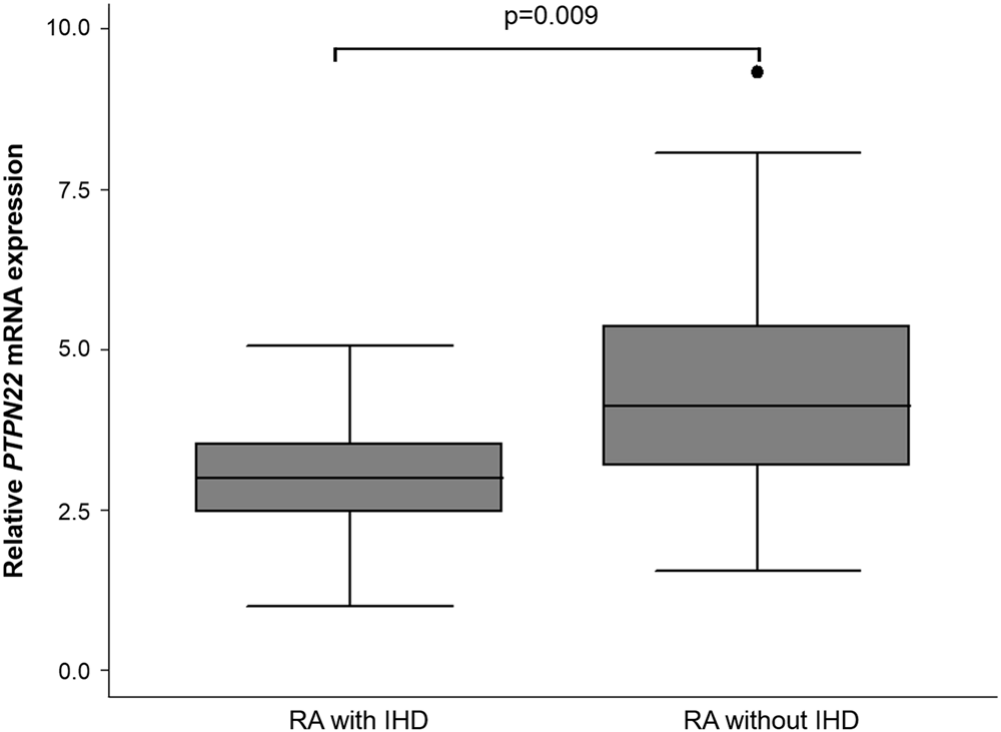
Decrease *PTPN22* mRNA expression in RA patients with IHD. Box plot showing the *PTPN22* mRNA expression, normalized to *beta-actin* and *GAPDH* expression, in patients with rheumatoid arthritis (RA) stratified according to the presence (n = 16) or absence of ischemic heart disease (IHD) (n = 71). P-value was adjusted for sex, age, and cardiovascular risk factors.

Interestingly, a significant association between *PTPN22* expression and ischemic heart disease (IHD) was observed in patients with RA, after and adjustment for sex, age at time of study, and CV risk factors (p = 0.009) (Fig. 3). In this regard, RA patients with IHD had a lower *PTPN22* expression (3.02 ± 1.11) than those without IHD (4.32 ± 1.57) (Fig. 3). In addition, an inverse correlation was disclosed between *CSK* expression in patients with RA and their C-reactive protein (CRP) levels at time of study (r = −0.26, p = 0.019). No significant differences were found between the *PTPN22* and *CSK* mRNA expression of patients with RA and other clinical characteristics, including disease duration, rheumatoid factor, anti-cyclic citrullinated peptide antibodies status, erosions, extra-articular manifestations, and CRP levels and erythrocyte sedimentation rate at RA onset.

### PTPN22 and CSK serum levels in RA and controls

The measurement of circulating levels of PTPN22 and CSK was assessed to provide supplementary reliable data between the expression of mRNA and the corresponding protein levels. No significant differences were found between patients with RA and controls in PTPN22 and CSK protein levels after adjustment by potential confounding factors. In this regard, PTPN22 levels were 450.59 ± 328.76 pg/ml in RA patients and 285.80 ± 259.70 pg/ml in controls (p = 0.20). Similarly, CSK levels were 39.02 ± 26.10 pg/ml in RA patients and 32.35 ± 24.53 in controls (p = 0.76).

## Discussion

Since an association between some *PTPN22* genetic variants and RA susceptibility was described^3–7^, functional analyses of *PTPN22* have been considered necessary to further understand the role of this gene in RA and other autoimmune diseases^1, 9^. Our study disclosed for the first time that two *PTPN22* genetic variants, rs2488457, located in the promoter region, and rs2476601, located in exon 14, down-regulate the transcription and function of *PTPN22* in patients with RA compared to controls, who have higher *PTPN22* expression. This was observed both when the *PTPN22* SNPs were analyzed individually or forming haplotypes. Our results confirm the trend found between *PTPN22* rs2488457 and *PTPN22* expression in Chinese RA patients^12^. Furthermore, Zhang *et al*. had previously shown that this SNP was associated not only with the development of an inflammatory disease known as Vogt-Koyanagi-Harada, but also with a decreased *PTPN22* expression, only assessed in healthy controls^18^. A potential influence between rs2488457 and ulcerative colitis was also described^19^. Regarding *PTPN22* rs2476601, it has been clearly shown that it is the main variant involved in the susceptibility to develop autoimmune diseases^1–3^, and due to its functional effect it is able to decrease the interaction between PTPN22 and CSK^14, 20^. No association between this SNP and *PTPN22* modulation had previously been shown^8, 21, 22^. However, *PTPN22* rs2476601 and also *PTPN22* rs2488457 have been recently proposed as potential cis-eQTLs in whole blood from RA patients^17^. This finding is in keeping with our results, supporting that these genetic variants may play an important role in the regulatory region of *PTPN22*. Based on our data, we feel that the decreased *PTPN22* expression in patients carrying the risk alleles of *PTPN22* rs2488457 and rs2476601 may lead to a pro-inflammatory status in RA, since T cells activation would not be inhibited by this gene. In fact, *PTPN22* deficiency significantly increased the severity of disease in a mouse model of RA^23^.

An important implication of PTPN22 in the development of atherosclerosis has previously been proposed^24^. In this regard, we disclosed for the first time a marked down-regulation of *PTPN22* gene in RA patients with IHD. Considering a previous study from our group which showed that *PTPN22* rs2476601 polymorphism was not directly associated with an increased risk of CV disease in RA patients^25^, we think that this association may be SNP-independent and could constitute an useful tool to identify RA patients with a higher CV risk.

We also demonstrated a significant decrease of *CSK* gene in peripheral blood from RA patients. A down-regulation of *CSK*, which acts as a tumor suppressor gene, has previously been associated with colon cancer^26^. However, the role of *CSK* gene in RA remains to be elucidated, even though it has been proposed as an autoimmunity risk factor^16^. Since a study demonstrated a decrease of inflammation in a rat model of RA injected with Csk virus^27^, we consider that the down-regulation of *CSK* may cause a high inflammatory response in our patients, contributing to RA progression. Finally, we observed that *CSK* rs34933034 and rs1378942 did not influence on *CSK* expression since its reduction in RA patients as compared to controls was independent of the polymorphisms. Therefore, the lower *CSK* mRNA expression shown in our RA patients when compared to healthy controls may be more related to the disease itself. The negative association of *CSK* mRNA expression with CRP, a systemic marker of inflammation, reinforces our hypothesis.

In summary, our study reveals for the first time that the risk alleles of *PTPN22* rs2488457 and *PTPN22* rs2476601 influence on the down-regulation of *PTPN22* in RA. This confirms the important role of *PTPN22* in RA. Furthermore, it indicates that *CSK* may also be considered as a genetic risk factor for the development of RA. The transcriptional suppression of the PTPN22/CSK complex, as a key regulator of the immune response, may have a noteworthy clinical relevance in patients with RA, playing an important role in disease progression. Compensatory mechanisms through other biologic pathways may be crucial to control RA.

## Subjects and Methods

### Patients and controls

For experiments involving humans and the use of human blood samples, all the methods were carried out in accordance with the approved guidelines and regulations, according to the Declaration of Helsinki. All experimental protocols were approved by the local Ethics Committees of Clinical research of Cantabria for Hospital Universitario Marqués de Valdecilla in Santander and of Madrid for Hospital Universitario de La Princesa. An informed consent was obtained from all subjects before being enrolled.

Peripheral blood samples were collected from 89 patients with RA who met the 1987 American College of Rheumatology (ACR) and also the 2010 ACR/European League Against Rheumatism criteria for RA^28, 29^ and 43 healthy controls recruited from Hospital Universitario Marqués de Valdecilla (Santander, Spain) and Hospital Universitario de La Princesa (Madrid, Spain). Supplementary Table 2 shows the main demographic and clinical characteristics of subjects enrolled in this study.

### Expression profiles of *PTPN22* and *CSK* genes

Total RNA from peripheral blood was isolated using NucleoSpin RNA Blood Midi kit (Macherey-Nagel) according to the manufacturer’s protocol, as we previously described^30, 31^. 1 μg of total RNA was reverse-transcribed using iScriptTM Advanced cDNA Synthesis Kit for RT- qPCR (Bio-Rad, Hercules, CA, USA). 20 ng of cDNA was used for quantitative real-time PCR (qPCR), using SsoAdvancedTM Universal SYBR® Green Supermix (Bio-Rad, Hercules, CA, USA). Primers for amplification of *PTPN22* (135 bp, UniGene ID Hs.535276), *CSK* (73 bp, UniGene ID Hs.77793), *beta-actin* (62 bp, UniGene ID Hs.520640) and *GAPDH* (117 bp, UniGene ID Hs. 544577) genes were acquired from PrimePCR Assays, Bio-Rad, Hercules, CA, USA. All samples were examined in triplicate and controls were included in each reaction. qPCRs were performed in a 7900 HT real-time instrument (Applied Biosystems, Foster City, CA, USA) following the conditions previously reported^31^. Amplicons of the different genes and a 50 base pairs DNA ladder (ThermoScientific) were electrophoresed on 2% agarose gel stained with GelGreen Nucleic Acid Gel stain (Biotium) to check the appropriate size of each amplicon (Supplementary Figure 1). *CSK* gene was expressed successfully in all samples. However, *PTPN22* was not detected in 2 out of the 89 patients enrolled. The relative *PTPN22* and *CSK* mRNA (messenger RNA) expression was analyzed by the comparative threshold cycle method, using *beta-actin* and *GAPDH* as housekeeping genes^30^. Normalized values were obtained for each sample and mean values were determined for each study group.

### *PTPN22* and *CSK* genotyping

Genomic DNA was extracted from peripheral blood samples using NucleoSpin Blood Kit (Macherey-Nagel) according to the manufacturer’s recommendations. Genotyping of the *PTPN22* rs2488457 (C > G), *PTPN22* rs2476601 (G > A)*, PTPN22* rs33996649 (C > T), *CSK* 34933034 (G > A) and *CSK* rs1378942 (A > C) was performed using TaqMan probes in a 7900 HT real-time instrument (Applied Biosystems, Foster City, CA, USA), as previously reported^32^. Negative controls were included to check the accuracy of genotyping. The linkage disequilibrium pattern of the *PTPN22* and *CSK* polymorphisms is displayed in Supplementary Figure 2.

### PTPN22 and CSK serum levels by enzyme-linked immunosorbent assay (ELISA)

PTPN22 and CSK serum levels were determined using commercial kits acquired from CUSABIO (Catalog Number: CSB-EL019036HU and CSB-EL006056HU, respectively), according to the manufacturer’s instructions. All samples were analyzed in duplicate.

### Statistical analysis

First, Student’s t test was used to compare differences in *PTPN22* and *CSK* mRNA expression between: patients and controls, patients and controls stratified according to their reference or risk allele for each SNP, as well as patients and controls stratified according to *PTPN22* and *CSK* haplotypes. Differences in PTPN22 and CSK serum levels between RA patients and controls were also assessed by Student’s t test. Results were expressed as mean ± standard deviation for each study group. Next, covariance analysis was performed in order to adjust the results by potential confounding factors, including sex, age at time of study, and cardiovascular (CV) risk factors (hypertension, dyslipidemia, smoking, diabetes and obesity)^33^. The association between *PTPN22* and *CSK* mRNA expression in RA patients and their clinical characteristics was evaluated using Student’s t test or Pearson partial correlation coefficients (r) as required, after adjusting for the confounding factors commented above. In all cases, p-values < 0.05 were considered statistically significant. Regarding genotyping, Hardy-Weinberg equilibrium (HWE) was checked using Χ^2^ test. The statistical power of the study is displayed in Supplementary Table 3. All these analysis were performed with STATA statistical software 12.0 (Stata Corp., College Station, TX, USA).

## Electronic supplementary material

Supplementary information
