# Supplementary material for: Protein tyrosine phosphatase non-receptor 22 and C-Src tyrosine kinase genes are down-regulated in patients with rheumatoid arthritis
Source: Sci Rep. 2017 Sep 5;7:10525. doi: 10.1038/s41598-017-10915-9 (PMC5585411; doi:10.1038/s41598-017-10915-9)

Protein tyrosine phosphatase non-receptor 22 and _C-Src tyrosine kinase_ genes down-regulated in patients with rheumatoid arthritis

Sara Remuzgo-Martínez, PhD[1,†], Fernanda Genre, PhD[1,†], Santos Castañeda, MD, PhD[2], Alfonso Corrales, MD, PhD[1], Pablo Moreno-Fresneda, MD[2], Begoña Ubilla, BSc[1], Verónica Mijares, BSc[1], Virginia Portilla, BSc[1], Jesús González-Vela, BSc[1], Trinitario Pina, MD, PhD[1], Gonzalo Ocejo-Vinyals, MD, PhD[3], Juan Irure-Ventura, BSc[3], Ricardo Blanco, MD,PhD[1], Javier Martín, MD,PhD[4], Javier Llorca, MD,PhD[5], Raquel López-Mejías, PhD[1,*], and Miguel A. González-Gay, MD,PhD[1,6,*].

Supplementary table 1. Differential *PTPN22* and *CSK* mRNA expression between healthy controls and RA patients according to their haplotypes.

	Haplotypes	mRNA expression controls vs RA patients (mean ± SD)	P-value
***PTPN22* Haplotypes**	CG	4.62 ± 1.55 vs 4.11 ± 1.60	0.89
**(rs2488457 and rs2476601)**	GA	4.76 ± 1.07 vs 3.80 ± 1.26	0.007
	GG	4.79 ± 1.71 vs 4.17± 1.76	0.63
***CSK* Haplotypes**	GA	3.13 ± 0.94 vs 2.47 ± 0.74	<0.0001
**(rs34933034 and rs1378942)**	GC	3.72 ± 1.10 vs 2.41 ± 0.78	<0.0001
	AC	3.14 ± 1.01 vs 2.69 ± 0.79	0.056

Supplementary table 2. Demographic and clinical characteristics of controls and RA patients enrolled in the study.

	Healthy controls, n=43	RA patients, n=89
Women, n (%)	26 (60.5)	68 (76.4)
Age at time of study (years, mean±SD)	50.5 ± 14.0	63.2 ± 11.6
Disease duration (years, mean±SD)	-	11.5 ± 10.5
Rheumatoid factor positive, n (%)	-	52 (58.4)
Anti-CCP antibodies positive, n (%)	-	55 (61.8)
Erosions, n (%)	-	44 (49.4)
Extra-articular manifestations*, n (%)	-	15 (16.9)
CRP (mg/l) at RA onset (mean±SD)	-	16.1 ± 20.6
ESR (mm/1st h) at RA onset (mean±SD)	-	34.5 ± 29.2
CRP (mg/l) at time of study (mean±SD)	2.4 ± 3.0	6.7 ± 8.8
ESR (mm/1st h) at time of study (mean±SD)	13.5 ± 11.9	19.2 ± 20.7
Ischemic heart disease, n (%)	0 (0.0)	16 (18.0)
Cardiovascular risk factors		
Hypertension, n (%)	7 (16.28)	47 (52.8)
Dyslipidemia, n (%)	12 (27.9)	50 (56.2)
Current smoking, n (%)	16 (37.2)	31 (34.8)
Diabetes, n (%)	0 (0.0)	10 (11.2)
Obesity, n (%)	8 (18.6)	31 (34.8)

Anti-CCP: anti-cyclic citrullinated peptide antibodies; CRP: C-reactive protein; ESR: erythrocyte sedimentation rate; RA: rheumatoid arthritis; SD: standard deviation.

*If patients experienced: nodular disease, Felty´s syndrome, pulmonary fibrosis, rheumatoid vasculitis, or secondary Sjögren´s syndrome.

Supplementary table 3. Statistical power analysis.

Group	Mean difference	Statistical power
Major allele homozygous	1 SD	0.99
	2/3 SD	0.87
Heterozygous or minor allele homozygous	1 SD	0.93
	2/3 SD	0.73

For estimating statistical power, a general scenario with 60% patients homozygous for the most frequent allele and 40% heterozygous or homozygous for the minor allele was considered. Then, two mean difference scenarios (mean difference equals to 1 SD and mean difference equals to 2/3 SD) were considered.

Supplementary figure 1. Expression of *CSK* and *PTPN22* in controls (a) and patients with rheumatoid arthritis (b). Amplicons of each gene and a 50 base pairs (bp) DNA ladder were electrophoresed on 2% agarose gel. *Beta-actin* (*ACTB*) and *GAPDH* used as housekeeping genes were also included.

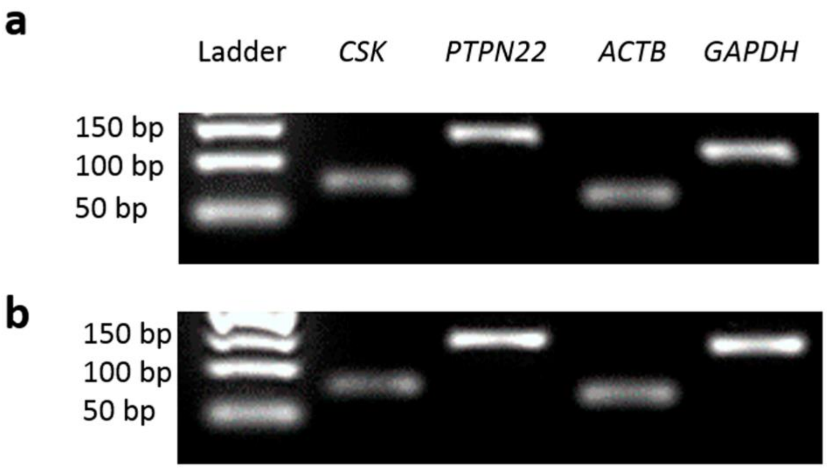

Supplementary figure 2. **Linkage disequilibrium (LD) plots (measured by r^2) of the *PTPN22* and *CSK* polymorphisms (in European population) tested in our study**. Data obtained by 1000 genomes and Haploview v.4.2 software. The LD between the polymorphisms studied is shown in a scale from minimum (white) to maximum (black).

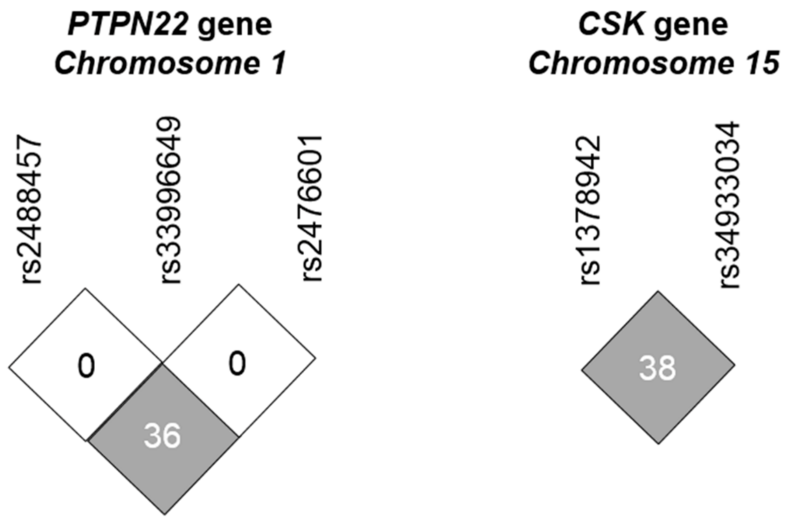

Supplement: Supplementary file 1 — Supplementary information [file 41598_2017_10915_MOESM1_ESM.pdf]
